# Organized Disassembly of Photosynthesis During Programmed Cell Death Mediated By Long Chain Bases

**DOI:** 10.1038/s41598-020-65186-8

**Published:** 2020-06-25

**Authors:** Alonso Zavafer, Ariadna González-Solís, Silvia Palacios-Bahena, Mariana Saucedo-García, Cinthya Tapia de Aquino, Sonia Vázquez-Santana, Beatriz King-Díaz, Marina Gavilanes-Ruiz

**Affiliations:** 1 Climate Change Cluster, University of Technology Sydney, Faculty of Science Building 4, Level 6 Corner of Thomas and, Harris St, Ultimo NSW 2007, Sydney, Australia; 2 Dpto. de Bioquímica, Facultad de Química, Conjunto E. Universidad Nacional Autónoma de México (UNAM). Ciudad Universitaria, 04510 Ciudad de México, México; 3 Instituto de Ciencias Agropecuarias, Universidad Autónoma del Estado de Hidalgo, Tulancingo, Hidalgo México; 4 Dpto. de Biología Comparada, Facultad de Ciencias, Universidad Nacional Autónoma de México (UNAM). Ciudad Universitaria, 04510 Ciudad de México, México

**Keywords:** Photosynthesis, Plant stress responses, Biochemistry, Plant sciences

## Abstract

In plants, pathogen triggered programmed cell death (PCD) is frequently mediated by polar lipid molecules referred as long chain bases (LCBs) or ceramides. PCD interceded by LCBs is a well-organized process where several cell organelles play important roles. In fact, light-dependent reactions in the chloroplast have been proposed as major players during PCD, however, the functional aspects of the chloroplast during PCD are largely unknown. For this reason, we investigated events that lead to disassembly of the chloroplast during PCD mediated by LCBs. To do so, LCB elevation was induced with *Pseudomonas syringae* pv. *tomato* (a non-host pathogen) or Fumonisin B1 in *Phaseolus vulgaris*. Then, we performed biochemical tests to detect PCD triggering events (phytosphingosine rises, MPK activation and H_2_O_2_ generation) followed by chloroplast structural and functional tests. Observations of the chloroplast, via optical phenotyping methods combined with microscopy, indicated that the loss of photosynthetic linear electron transport coincides with the organized ultrastructure disassembly. In addition, structural changes occurred in parallel with accumulation of H_2_O_2_ inside the chloroplast. These features revealed the collapse of chloroplast integrity and function as a mechanism leading to the irreversible execution of the PCD promoted by LCBs.

## Introduction

The simplest plant sphingolipids are long chain bases (LCBs), which have a signaling role during stomata closure, cold response and programmed cell death or PCD (immunity associated)^1–9^. In the case of PCD associated with pathogen immunity, there has been indication of a transient rise of long chain bases (LCBs): sphinganine^10,11^ and phytosphingosine^12^. Plants express PCD during differentiation, development, senescence, and immunity, but it is the latter in which LCBs have been demonstrated as transducers. The Hypersensitive Response (HR) is a PCD form which is a common and powerful mechanism of plant defense against pathogens^13,14^. HR is visualized as a dry tissue spot developed at the entry site of certain pathogens, wherein cells launch a death program. This prevents the supply of nutrients to the biotroph intruder, avoiding its dissemination^15^. LCBs have been suggested as second messengers in the HR-PCD involved in forms of plant immunity. Interestingly, some other signaling events in this PCD transduction pathway have also been described: generation of reactive oxygen species generation (ROS)^11–18^, MPK6 activation^11^ and a calcium surge^10^, but their integration as a whole chain of events remains to be established.

Some studies on PCD related to immunity have shown effects on the chloroplast: (1) PCD evoked by *Pseudomonas syringae* through MPK6 intermediation results in an inhibition of CO_2_ fixation and H_2_O_2_ accumulation in chloroplasts^19^; (2) PCD elicited by inhibition of the electron transport beyond Q_A_ in photosystem II is related to systemic immunity^20^; (3) PCD produced by an avirulent strain of *Pseudomonas syringae* was suggested to be influenced by disassembly of the photosynthetic complexes, probably due to ROS generation in the chloroplast^21^. Moreover, it has been recognized that photosynthetic activity is interrupted upon pathogen infection^22–24^. All this diverse information suggests that the chloroplast and its photosynthetic activity are involved in the PCD process.

In parallel, evidences dealing with the PCD-HR mediated by LCBs suggest an association with the chloroplast: (1) PCD elicited by fumonisin B1 (FB1, a mycotoxin that evokes LCB accumulation^25^) is light-dependent^16^; (2) PCD induced by FB1 and mediated by MPK6^11^ promotes extensive chloroplast damage and induces H_2_O_2_ formation inside the chloroplasts^11,19^. However, a study of the chloroplast and its function during HR-PCD that is mediated by LCBs has not been specifically addressed.

Therefore, these and other unknown events dealing with the chloroplast need to be identified and positioned in a single sequence in order to establish a clear functional context. For this reason, we have induced PCD in *Phaseulus vulgaris* using two LCB eliciting treatments: FB1 and a non-host pathogen that induces HR-PCD. Then, we explored if LCB accumulation took place in both treatments and measured some biochemical responses such as reactive oxygen species formation and MAP kinases activation. Then, we analyzed the chloroplast ultrastructure and the functioning of its light-dependent reactions. Here, we provide direct evidence that there is a direct organized and irreversible collapse of the chloroplast that leads to the PCD mediated by LCBs.

## Materials and methods

### Chemicals

Fumonisin B1, sphinganine, phytosphingosine, myelin basic protein (MBP), α-casein bovine milk, and calf-thymus histone III were purchased from Sigma Chemicals (St. Louis Mo). D-erythro C20-sphingosine was purchased from Matreya Inc. (Pleasant Gap, PA). Silwet L-77 was obtained from Chemtura Corporation S. de R.L. de C.V. (Mexico City, México). γ-[^32^P]-ATP (Easy Tides 3000 Ci/mmol-10 mCi/ml, pH 7.6 was purchased from PerkinElmer (Austin, Tx).

### Biological material

*Phaseolus vulgaris* var. Canario (common bean) plants were grown in agrolite, watered with Hoagland solution and maintained under a natural photoperiod at 28 °C in a greenhouse. *Pseudomonas syringae* pv. *tomato* DC3000 avr*RPM1* (Pst), a strain that elicits a defense response in *Phaseolus*, was cultured in solid B King medium with rifampicin and tetracycline at 50 µg/ml. Fresh cultures were resuspended in 10 mM MgCl_2_ and then leaf-infiltrated.

### *In planta* treatments

MgCl_2_, Silwet L-77, FB1, sphinganine (SN), salicylic acid (SA) and *Pseudomonas syringae* were infiltrated in fully expanded leaves attached to healthy 4–6-week-old *Phaseolus* plants. Infiltration was done on the leaf abaxial surface in 4–6 points per leaf with a needleless syringe^26^. Every point was infiltrated with 20 µL of the following compounds: 10 mM MgCl_2_ (as control), 0.05% Silwet L-77 (as control), 5–50 µM FB1 dissolved in 10 mM MgCl_2_, 40 µM SN dissolved in 0.05% Silwet L-77, 1 mM salicylic acid (SA), pH 7 and a suspension of *Pseudomonas syringae* pv. *tomato* at 1 × 10^5^ to 1 × 10^8^ CFU (colony forming units) per ml as indicated.

Samples at the infiltration sites were taken in the interval from 0 to 48 h after treatments as indicated. Treatments with MgCl_2,_ Silwet L-77, FB1, Pst, SN or SA were performed while the leaves were attached to the plant and then all measurements were carried out. A photographic record of the evolution of leaves upon different treatments was followed.

### Electrolyte leakage assay

Leaf disks of 1.0 cm diameter containing the infiltration points were cut at every infiltration time, weighed and electrolyte leakage was determined with a conductimeter Conmet1, Hanna Instruments (Woonsocket, RI)^27^. Briefly, leaf disks were submerged in double distilled water under moderate stirring and medium conductance was measured at several times. Electrolyte leakage was expressed following the formula ES = EC1/EC2 × 100, where ES corresponds to final conductivity of the sample, E1 corresponds to the electrical conductivity measured at 24, 48 and 72 h and EC2 corresponds to the conductivity measured at the end of the experiment when leaf disks were boiled to release total electrolytes into the medium.

### Determination of MAPK activity

 *Phaseolus*  leaves were infiltrated with MgCl_2_, Silwet L-77, FB1, SN, SA or *Pseudomonas syringae* pv. *tomato* during the indicated times and immediately frozen and maintained at −70 °C. Then, at periods no longer than one week, leaves were homogenized to obtain the soluble fractions and in-gel MAPK activity was performed supplementing MBP to the polyacrylamide matrix to serve as phosphorylation substrate using γ-[^32^P]-ATP. This assay was performed using α-casein and type III histone as negative controls to identify specific MAPK phosphorylating activity.

### Determination of H_2_O_2_*in situ*

Leaf disks of 1.0 cm diameter containing the infiltration treatments were immediately submerged in a solution of 3,3-diaminobenzidine (DAB)^28^. The H_2_O_2_ in the leaf tissue was visualized by a brown-reddish precipitate. Images were photographed (Moticam 2300, 3.0 M Pixel USB 2.0) coupled to a dissection microscope using the software Motic Images Plus 2.0 ml (Motic China Group www.motic.com).

### Determination of H_2_O_2_ in solution

Total levels of H_2_O_2_ were determined in the infiltrated tissue. Briefly, leaf disks of 1.0 cm diameter were sectioned from the leave sites infiltrated with MgCl_2_, FB1 or Pst and immediately homogenized and centrifuged. Aliquots from the supernatant were rapidly withdrawn to determine the ferric-xylenol orange complex^29^.

### Determination of LCBs

Extraction of LCBs was done according to Markham *et al*.^30^, starting from a microsomal fraction equivalent to 1.0 mg of fresh weight tissue. Briefly, the membrane fraction was supplemented with 2 nmol of *D*-erythro sphingosine as internal standard, 1 ml dioxane, 1.0 ml of 10% Ba(OH)_2_, and incubated at 110 °C during 16 h. Then 2 ml of 2% ammonium sulphate and 2 ml of diethyl ether were added. The organic phase was evaporated under a nitrogen flow until dry and the residue used for LCB determination. This was done by LCB derivatization with NDA (naphtalen-2,3-dicarboxy dialdehyde)^31^. Once derivatized, the samples were analyzed with a Shimadzu HPLC system with an UV detector (Mod. SPD-10AV).

### Protein determination

This was done according to Bradford (Bio-Rad, http://www.bio-rad.com/) using the Bio Rad Kit according to the manufacturer instructions.

### Determination of chlorophyll *a* fluorescence

This was performed *in planta* at the infiltration sites as follows: The leaf areas exposed to the treatments at the indicated times were dark-adapted for 10 min and then illuminated with continuous light (650 nm peak wavelength, 2800 µmol photon m^−2^ s^−1^). Chlorophyll *a* fluorescence was recorded during 1 s. The measurements were taken at infiltration times of 0, 8, 12, 24 and 48 h. Fast rise of the chlorophyll *a* fluorescence was measured with a Multifunctional Plant Efficiency Analyzer (M-PEA) (Hansatech Instruments Ltd., Norfolk, England).

### Determination of P700/P700^+^

Simultaneously with the measurements of chlorophyll *a* fluorescence, plastocyanin (PC), P700 and ferredoxin reduction/oxidation were measured using the 820 nm transmission change (far red light 820 nm peak wavelength) with MPEA (Hansatech Instruments Ltd., Norfolk, England).

### Determination of chloroplast ultrastructure

Leaf disks of 0.5 cm diameter were cut from the leaves infiltrated with MgCl_2_ (as control), FB1 solution or *Pseudomonas syringae* pv. *tomato* suspension. After the indicated times, the leaf disks were fixed in 100 mM phosphate buffer (pH 7.2) and 3% glutaraldehyde (v:v) at 4 °C, dehydrated and embedded in epon:polypropylene oxide. Sections of 80 nm were contrasted with uranyl acetate and lead citrate. Samples were observed with a Jeol 1200 EXII electron microscope (Jeol Ltd., http://www.jeol.com/) operated at 60 kV. Images were processed with Photoshop imaging software, version 8.0.1 (Adobe Systems, http://www.adobe.com/products/photoshop/family/).

### Determination of chloroplast autofluorescence

Infiltrated leaf disks were fixed as for the ultrastructural analysis, but thin sections from the disks were observed in a confocal microscope Olympus FV1000 using a wavelength excitation of 635 nm and a wavelength emission of 664 nm for chloroplast autofluorescence.

### Statistical analysis

All the results shown were the average values obtained from at least three independently grown/processed lots of plants. Statistical analyses were processed using Microsoft Excel 2007, SPSS 17 and Origin 7.0 as detailed in every experiment. All images were processed using ImageJ 1.z^32^.

## Results

### FB1 and *Pseudomonas syringae* pv. *tomato* induce similar responses associated to a PCD-HR in *Phaseolus vulgaris*

**Figure 1:**
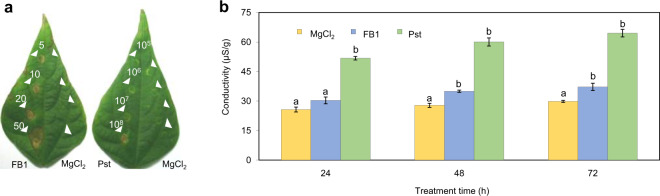
FB1 and Pst produce common PCD-HR macroscopic responses in *Phaseolus* leaves. (**a**) PCD-HR lesions. Leaves were infiltrated *in planta* with different concentrations of FB1 and Pst. Leaf at left shows points of infiltration with 1, 5, 10 and 20 μM FB1 as indicated. Leaf at right shows points of infiltration with 1×10^5^, 1×10^6^, 1×10^7^, 1×10^8^  CFU/ml Pst as indicated. Arrows at the  right side of both leaves show the infiltration of 10 mM MgCl_2_. Image shows lesions recorded after seven days of infiltration. Experiments were n > eight technical repeats from at least three biological replicates. (**b**) Electrolyte leakage. *Phaseolus* leaves were infiltrated *in planta* with 10 μM FB1 or 1×10^8^ UFC/ml Pst at the indicated times and infiltrated areas were cut, weighed and subjected to solute leakiness determination. Average values of n > eight technical repeats from three biological replicates ± standard error (s.e.m.). A mean comparison using a one-way ANOVA was done with a Tukey post-hoc test with *p* < 0.05. MgCl_2_ was used as reference for comparison at each time point. ^a^ No statistical difference between the control groups (MgCl_2_). ^b^Statistical differences between MgCl_2_ and FB1 or Pst treatments.

In order to confirm that *Phaseolus* was FB1-sensitive as it occurs in many, but not all species^33,34^, leaves were infiltrated with 5 to 50 μM FB1 (Fig. 1a). It was observed that the toxin produced a delimited spot the size of which was concentration-dependent (Fig. 1a, left leaf) and started to be visible 24–48 h after 10 μM FB1 infiltration. Since non-host species can produce a hypersensitive response (HR) as part of a defense reaction, we infiltrated 10^5^–10^8^  CFU/ml of a suspension from *Pseudomonas syringae* pv. *tomato* (Pst), a non-host pathogen, to bean leaves. Figure 1a (right leaf) shows that Pst suspension developed a necrotic zone the size of which was pathogen concentration-dependent and that was visible at 18–24 h after infiltration. Dead tissue remained circumscribed to the site of inoculation with FB1 or Pst, as it occurs in the classical HR and not in necrosis processes. As a control, infiltration of MgCl_2_ developed only a light circle caused by the slight wound (Fig. 1a, right side in both leaves). In order to corroborate that the FB1 and Pst treatments were inducing cell death, solute leakage was measured. Figure 1b shows that both, toxin and pathogen induced electrolyte release, but the kinetics and extent were faster and higher, respectively, with the Pst treatment as compared to the one elicited by the toxin.

### Elevation of LCBs

**Figure 2:**
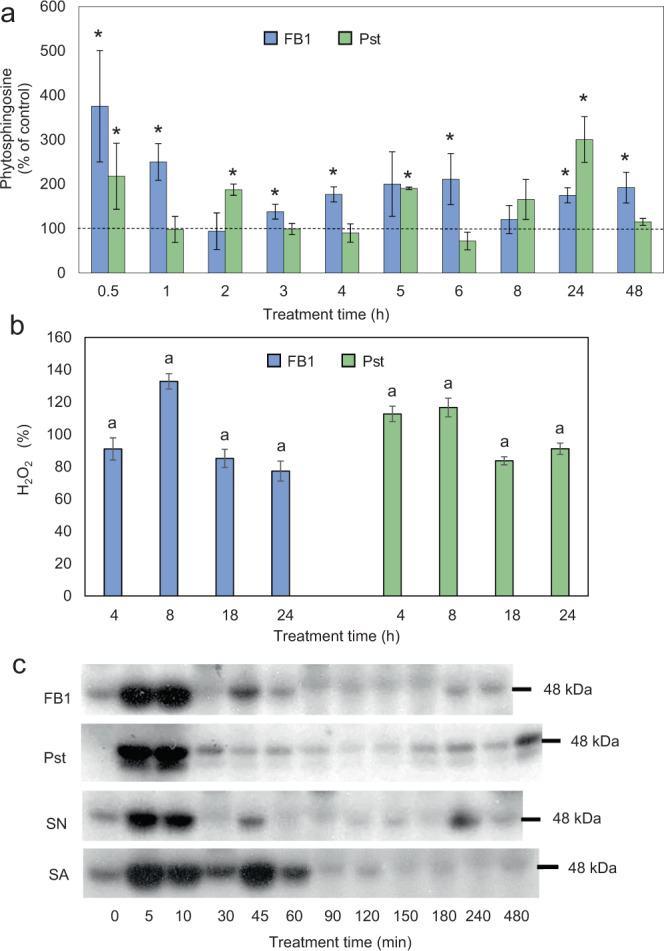
Pst and FB1 produce molecular responses associated to the PCD-HR mediated by LCB accumulation. (**a**) Long chain base levels. *Phaseolus* leaves were infiltrated *in planta* with 10 μM FB1 or 1×10^8^ CFU/ml Pst and at the indicated times, infiltrated leaf sections were used to isolate microsomal fractions to quantitate phytosphingosine levels. MgCl_2_-infiltrated leaves were used as controls and processed exactly in the same way that the FB1- or Pst-infiltrated leaves. The results are expressed in terms of the phytosphingosine levels as compared to the respective MgCl_2_-infiltrated sample (100%), processed at every time. Average values of n > six technical repeats from three biological replicates ± standard error (s.e.m.). A mean comparison using a one-way ANOVA was done with a Tukey mean difference plot post-hoc test with *p* < 0.05. Leaves infiltrated with MgCl_2_ were used as reference for comparison at each time point. (**b**) H_2_O_2_ levels. *Phaseolus* leaves were infiltrated *in planta* with 10 mM MgCl_2_, 50 μM FB1 or 1×10^8^ CFU/ml Pst and at the indicated times the infiltrated tissue was processed to determine total H_2_O_2_ levels in solution. The values with MgCl_2_ were considered as 100%. One way ANOVA and a posthoc Tukey with *p* < 0.05.) was performed. (**c**) MAPK activation. *Phaseolus* leaves were infiltrated *in planta* with 40 μM FB1, 1×10^8^ CFU/ml Pst, 40 µM sphinganine (SN) or 1 mM salicylic acid (SA); then, at the indicated times, leaf sections were cut and soluble protein fractions were obtained to determine in-gel MBP phosphorylation to estimate MAPK activity. Complete time courses and infiltration with MgCl_2_ or Silwet L-77 as controls were performed and are shown in Supplemental Fig. S2. Experiments are representative of at least three biological replicates.

FB1 elicits PCD and defense reactions through the elevation of LCBs as second messengers^11,16^. However, elevation of some sphingolipid species could not be detected in other non-host case^8^. Since in our system FB1 could be inducing  a PCD related to an increase in endogenous LCBs, we measured LCB levels in the FB1- and Pst-infiltrated leaves. Figure 2a shows the determination of endogenous phytosphingosine, a LCB, upon FB1 or Pst infiltration. We observed phytosphingosine increases of 1.5 to 3-fold along the infiltration time course with either treatment. Although the kinetics and maximal extent of the LCB elevation were not identical for FB1 and Pst, there were coincident times of phytosphingosine rise (0.5 and 24 h), showing a 2- to 3-fold increase over the LCB levels measured in control leaves. These results indicated that both treatments induced LCB elevation in *Phaseolus* leaves.

### H_2_O_2_ generation

Another known response in plant immunity is the early generation of H_2_O_2_^17,35,36^. For this reason, we measured this reactive oxygen species with a soluble assay in both PCD inducing treatments (Fig. 2b). The results showed that FB1 and Pst treatments induced an apparent significant generation of H_2_O_2_ at 8 h upon FB1 infiltration and at 4 and 8 h upon Pst infiltration. However, statistical significance was not supported by tests (see Fig. legend).

### MAPK activation

MAPK activation has been demonstrated to be downstream LCB surge in Arabidopsis PCD^11^. In order to get further support to the idea that the two treatments (FB1 and Pst) that induced PCD-HR were fully comparable in terms of the LCB elicitation, we investigated the possible activation of MAPKs upon FB1 and Pst infiltration of *P. vulgaris* leaves (Fig. 2c). In addition, we tested MAPK activation upon infiltration of SN, a LCB that elicits PCD-HR and that is precursor of phytosphingosine^11^. We detected the activation of a 48 kDa MAPK with the three treatments (Fig. 2c). The MAPK nature of the phosphorylation was corroborated by the specificity of the phosphorylated substrate i.e., myelin basic protein but not of histone or casein (Supplementary Fig. S1a,b) and the recognition of the 48 kDa band by antibodies directed against two typical MAPK, ERK1 and ERK2, and to their phosphorylated forms (Supplementary Fig. S1c). MgCl_2_ infiltration showed an activation of the same MAPK at 5 and 10 min that probably involved the slight wound effect due to the infiltration procedure (Supplementary Fig. S2a). While FB1 and SN showed a MAPK activation at 45 and 240 min, Pst displayed a sustained activation as long as 480 min after infiltration (Fig. 2c, Supplementary Fig. S2a–d). The molecular mass of the detected MAPK was similar to the AtMPK6 from *Arabidopsis thaliana* and to the NtSIPK from *Nicotiana tabaccum*^37,38^. The band, strongly phosphorylated in the presence of FB1, Pst and exogenous SN was dramatically labeled upon SA treatment (Fig. 2c, Supplementary Fig. S2a–e), a recognized mediator in plant immunity and an orthologue of MPK6 in Arabidopsis^37,38^. These results indicated that a MAPK commonly associated with defense responses was activated in conditions in which an elevation of an endogenous LCB occurs upon *Phaseolus* exposure to Pst.

### H_2_O_2_ accumulation at the lesion

**Figure 3:**
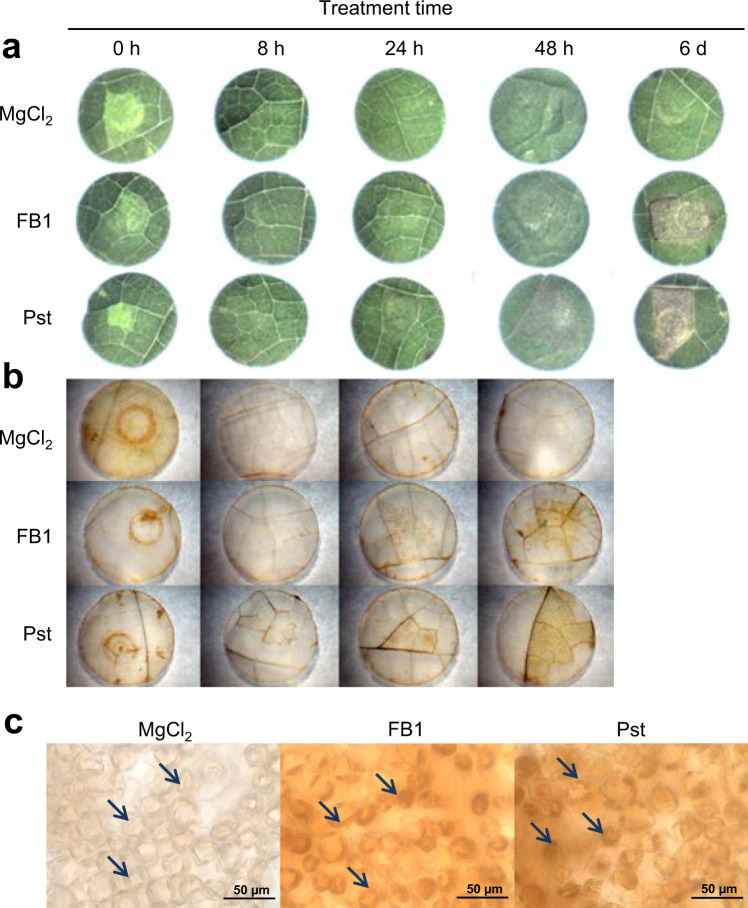
The PCD-HR lesions produced by FB1 and Pst treatments contain chloroplasts with H_2_O_2_ inside. (**a**) Time progression of the PCD-HR lesion. Leaves were infiltrated *in planta* with 10 mM of MgCl_2_, 10 μM of FB1 or 1×10^8^ of CFU/ml of Pst, then leaf disks with the infiltrated regions were cut and pictures taken at the indicated times. (**b**) *In situ* generated H_2_O_2_. Experiment was as in A, but leaf disks were subjected to the DAB reaction. (**c**) Magnification of the tissue with positive DAB reaction. Labeling of chloroplasts with the brown-reddish precipitate containing H_2_O_2_ is indicated by the arrows. The experiments shown are representative of four biological replicates.

We explored whether FB1 and Pst promoted *in situ* H_2_O_2_ accumulation in the infiltrated tissue by using DAB staining (Fig. 3). The lesion induced by either FB1 or Pst was clearly visible at 24 h in the case of Pst and at 48 h in the case of FB1 (Fig. 3a). After direct infiltration, *in situ* H_2_O_2_ formation was detected upon MgCl_2_, FB1 or Pst immediately after inoculation (0 h) (Fig. 3b). Between 18 and 24 h after infiltration, we detected H_2_O_2_ accumulation in both treatments. The brown precipitate progressively augmented at 48 h. These results confirmed that both FB1 and Pst shared as a common feature the H_2_O_2_ accumulation. No H_2_O_2_ stain was observed in the control treatment.

### Accumulation of H_2_O_2_ in the chloroplast is induced by treatments with FB1 or Pst

We examined the DAB stained tissues under the microscope in order to establish the location of the precipitate accumulation. In both experimental treatments, H_2_O_2_ accumulated in the chloroplast (Fig. 3c, central and right panels) but it was absent in the control leaves (Fig. 3c, left panel). This suggests that the possible origin of the accumulated H_2_O_2_ in FB1 and Pst treatments is the chloroplast.

### Chloroplast fluorescence is compromised upon FB1 or Pst treatments

**Figure 4:**
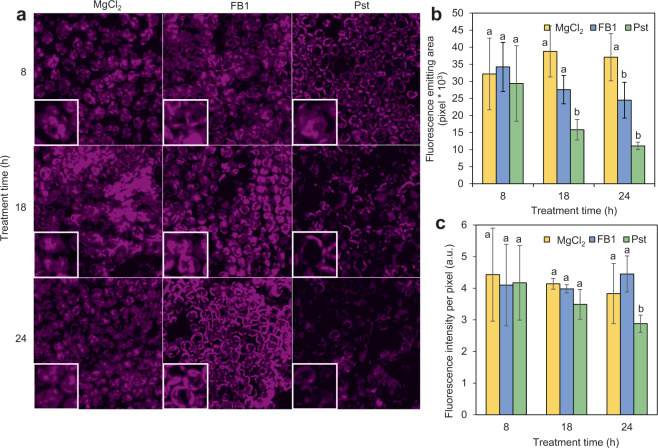
FB1 and Pst produce a loss of chlorophyll fluorescence. (**a**) Chloroplast fluorescence. *Phaseolus* leaves were infiltrated *in planta* with 10 mM MgCl_2_, 10 μM FB1 or 1×10^8^ CFU/ml Pst. At the indicated times, sections of the infiltrated tissue were analyzed by confocal microscopy. Fluorescent signal is shown in magenta color. (**b**) Average value of the fluorescence emission area. (**c**) Average value of the fluorescence intensity per pixel. The images shown are representative of three biological replicates.

H_2_O_2_ could be formed in the chloroplast as a result of impairment of the photosynthetic function. Our first approach to explore the functional status of the chloroplast treated with FB1 or Pst was to examine chloroplast structure using its autofluorescence by confocal microscopy (Fig. 4). Figure 4a shows images recorded at 8, 18 and 24 h after MgCl_2_, FB1 or Pst treatments. As it can be observed, chloroplasts from *Phaseolus* leaves that were MgCl_2_-infiltrated showed similar morphology, distribution and emission of chlorophyll fluorescence throughout this time course. The FB1 treated plants (Fig. 4a) showed an apparent decrease in the chlorophyll fluorescence signal at 24 h, but the graph in Fig. 4b shows that dispersion of data gave no significant difference between the control and FB1 fluorescence intensity values at 24 h. In contrast, Pst-treated plants (Fig. 4a, right column) showed a clear decrease of the fluorescence emission at 18 h (Fig. 4b). This decrease was more evident at 24 h when the signal was very low. These results can be explained by impairment of the photosynthetic activity of the treated leaves. To analyze this hypothesis further, the fluorescence emitting area and the fluorescence intensity per pixel were measured. While the first parameter correlates with the number of functional chloroplasts (Fig. 4b), the latter is an indication of the chloroplast fitness to perform photosynthetic activity per area unit (pixel) (Fig. 4c). Both parameters were unchanged in the chloroplasts from control plants. In the case of FB1-treated plants, a significant decay in the fluorescence emitting area was observed after 24 h (30% lower than in MgCl_2_-treated plants) (Fig. 4b). In Pst-treated plants, a clear decay in the fluorescence emitting area was observed at 18 h (64% lower as compared to the MgCl_2_-treated plants), with no significant decrease at 24 h (70% lower than MgCl_2_-treated plants) (Fig. 4b). However, neither FB1- or Pst-treated plants showed a significant decay in the fluorescence intensity per pixel as compared to the control (Fig. 4c). Thus, in the case where the photosynthetic activity was compromised, it could primarily be associated to a decrease in the number of functional chloroplasts with FB1 and or Pst, rather than with an overall decay in the activity of each chloroplast.

### Early and similar impairments of the photosynthetic activity are caused by FB1 and Pst treatments

**Figure 5:**
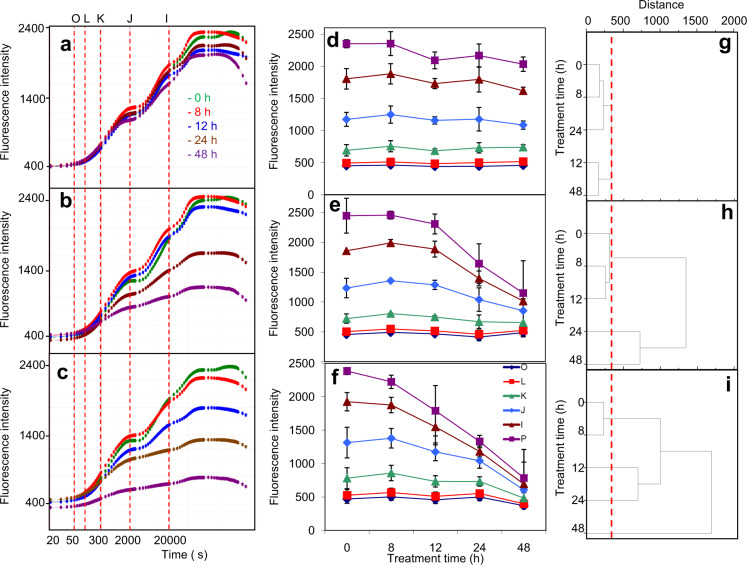
FB1 and Pst produce a decrease of chlorophyll *a* fluorescence upon treatments with FB1 or Pst. Chlorophyll *a* fluorescence was measured *in planta* in the infiltrated zones of the leaves. Panels a-c show the average OJIP transients for leaves infiltrated with (**a**) 10 mM MgCl_2_, (**b**) 10 µM FB1 or (**c**) 1×10^8^ CFU/ml Pst at the indicated times. Panels (**d–f**) show the average value of each fluorescent step (OLKJIP) of leaves infiltrated with (**d**) 10 mM MgCl_2_, (**e**) 10 µM FB1 or (**f**) 1×10^8^ CFU/ml Pst, respectively, at the indicated times. Curves and values represent the average ± SD of four biological replicates. Step O was measured at 50 μs, L at 150 μs, K at 300 μs, J at 2000 μs, I at 30,000 μs and P when fluorescence reached its maximum. (**g–i**) Statistical differences for each time point were estimated by multivariate hierarchical clustering analysis for MgCl_2_ (**g**), FB1 (**h**) and Pst (**i**). Values of the O, L, K, J, I and P were used to estimate the Euclidian distance and were clustered by group average. The longer the distance in a cluster, the more different two time points were. To estimate the threshold of significance, the maximum distance in MgCl_2_ data set was used as reference. Red dotted line describes the threshold for significance, in such case a node situated at the left of the red line is considered not significant. OJIP plots were constructed from data acquired with the HandyPEA instrument as described under Materials and methods section and processed by the Biolyzer 3.0 software, Bioenergetics Laboratory, Geneva, Switzerland.

While autofluorescence measurements were a good indicator of photosynthetic impairment, we explored the effect of FB1 and Pst on the photosystem II (PSII) activity. Thus, we measured the chl *a* fluorescence induction curve (OJIP curve) between 0 h (pre-infiltration sampling) and 48 h (0, 8, 18, 24 and 48 h). Figure 5a–c shows the average curves of fluorescence emission. No significant changes in the control leaves (MgCl_2_-infiltrated, Fig. 5a) were found as indicated by the low scattering of the curves, especially in phases O-I, at all infiltration times under study. This implied that the MgCl_2_ infiltration produced only slight perturbations in the photosynthetic activity at the time span of 48 h. However, Fig. 5b,c clearly showed, respectively, that the FB1 and Pst infiltration produced impairments in the photosynthetic activity as a function of the treatment time.

We compared the change in the fluorescence intensity at O, L, K, J, I and P steps as a function of the time of exposure to MgCl_2_ (Fig. 5d), FB1 (Fig. 5e) or Pst (Fig. 5f). Figure 4e shows that the earliest apparent decrease on chl *a* fluorescence occurred at 12 h with FB1 and at 8 h with Pst. In the case of prompt chlorophyll fluorescence, the first statistically significant decrease took place at 24 h for FB1-treated plants while with Pst treatment it occurred after 12 h (Fig. 5f). JI and IP phases presented the major changes in both treatments at 24 and 48 h but were more pronounced with Pst. Statistical significance was performed for all curves by multivariate hierarchical clustering analysis (Fig. 5g–i). The decrease in the early decay of the J step with Pst can be interpreted as a decrease in the capacity to perform QA reduction (photochemical phase of PSII), meanwhile the decrease in the JI and IP phases could reflect loss of electron transfer between PSII to Photosystem I (PSI). Therefore, the most affected part of the photosynthetic process that was associated to LCB surges was the electron transport.

**Figure 6:**
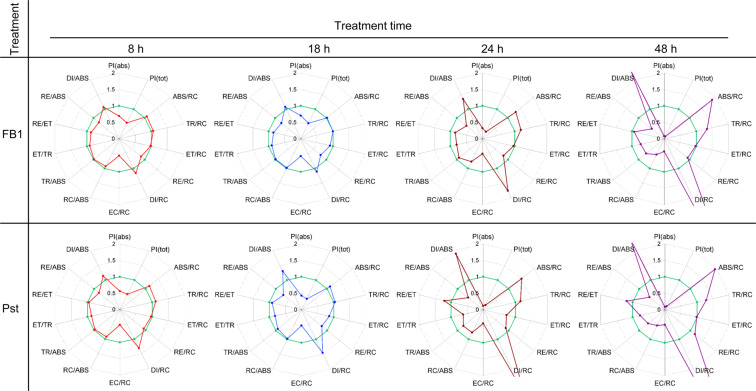
FB1 and Pst alter the same OJIP-test parameters. Average OJIP curves shown in Fig. 5 were used to calculate OJIP-test parameters. All parameters were normalized to the control value (pre-infiltrated plants) and represented in the radar plots shown. PI(abs) refers to photosynthetic performance index on absorption bases. PI(tot) refers to total photosynthetic performance index. ABS/RC refers to absorption per active PSII reaction center. TR/RC refers to trapping probability per active PSII reaction center. ET/RC refers to the probability of electron transport beyond Q_A_ per active PSII reaction center. RE/RC refers to the probability of an electron to be used in the reduction of final acceptors per active PSII reaction center. DI/RC refers to proportional energy dissipated by heat or fluorescence per active PSII reaction center. EC/RC refers to the rate of turnover by electron carriers per active PSII reaction center (which is estimated as the complementary area over the OJIP curve). RC/ABS is reciprocal of ABS/RC (see above). TR/ABS refers to the quantum yield for primary photochemistry. ET/TR refers to the yield for electron transport. RE/ET refers to the probability of an electron traveling through the electron transport chain to be used in the reduction of end acceptors. RE/ABS refers to the total quantum yield of photosynthesis. DI/ABS refers to quantum yield for energy dissipation. Note that OJIP-test parameters were calculated from average OJIP curves in Fig. 5, hence the statistical significance of these parameters is based on Fig. 5g–i. The OJIP parameters shown in the eight radar plots were obtained from the data in Fig. 5 using the Biolyzer 3.0 software, Bioenergetics Laboratory, Geneva, Switzerland.

In order to know the state of the energy flow parameters, OJIP curves were analyzed by using the JIP-test model^39,40^. These parameters are presented in the form of radar plots (Fig. 6) and were calculated using  the average curves in Fig. 5a–c. The comparison of data from leaves treated with FB1 or Pst for every time, i.e. 8 h, 18 h, 24 h and 48 h (Fig. 6) revealed a striking similarity in the magnitude and pattern of effects on JIP-test parameters. In the leaves exposed to FB1 and Pst for 18 h (Fig. 6), the total performance photosynthetic index (PI_tot_) parameter was the most negatively affected. According to the PI_tot_ calculation, the decrease was mainly due to a decrease in the absorption per reaction center component (ABS/RC), i.e. an alteration of the ratio of the absorbed energy and the number of functional reaction centers. Therefore, there was a decrease in the number of functional PSII units. This result was in agreement with the information given by the images of confocal microscopy (Fig. 4a,b). In addition, the values of the yield of energy dissipation per reaction center (DI/ABS and DI/RC, respectively), dramatically enlarged upon 18 h of FB1 or Pst exposure.

Another photosynthetic parameter negatively affected after 18 h of exposure to FB1 or Pst was the electron carrier pool per reaction center (EC/RC). Such result was expected as the JI and IP phases were the most impaired. Based on the JIP algorithm analysis, increases in LCB were due to either the loss in the total pool of electron carriers or to the compromised turnover capacity of PSII, reducing the carrier molecules.

Together, these results suggest that both experimental treatments decreased the number of functional PSII reaction centers and the electron carriers.

### Disconnection between PSII and PSI is promoted by FB1 or Pst treatments

**Figure 7:**
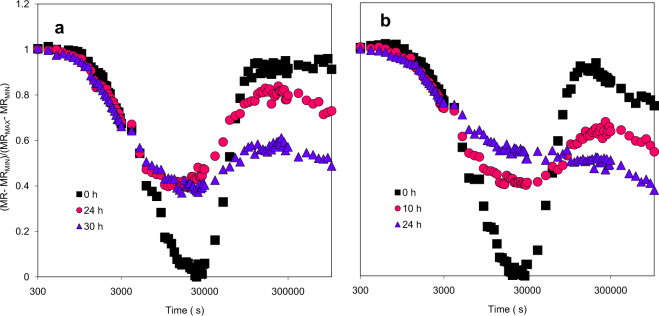
FB1 and Pst affect connectivity between PSII and PSI in leaves infiltrated with FB1 or Pst. Modulated reflection (MR) of 820 nm light was measured *in planta* to determine the redox state of P700 in the infiltrated regions of the plants. MR kinetics from leaves infiltrated with (**a**) 10 µM FB1 or (**b**) 1×10^8^ CFU/ml Pst at the indicated times. Values were normalized to the pre-infiltration values. MR signal was collected in parallel to the OJIP transients (Fig. 5) by exciting the sample with 3000 µmol of photons/m^2^/s. Curves are representative of three biological replicates.

The OJIP-test suggested that one of the major impairments of photosynthesis during PCD elicited by LCBs was the flow of electron carriers per active reaction center. This can be directly determined by measuring the linear electron flow between PSII and PSI by reflectance changes at 820 nm. Modulated light reflection (MR) is an approach to assess the oxidation state of P700 + and PC + ^41–43^. MR was measured in parallel to the chl *a* fluorescence at pre-infiltration (0 h) and 10, 24 and 30 h after MgCl_2,_ FB1 or Pst treatment (Fig. 7). MR experimental curves were normalized to the values at 0 h (pre-infiltration) for each condition to simplify the comparison.

MR determinations of leaves infiltrated with MgCl_2_ and with 0 h exposure to FB1 (Fig. 7a) or to Pst (Fig. 7b) leaves showed that plants behaved in similar manner. The first phase, a fast phase (MR_fast_), occurring in approximately a time span of 0.001 and 0.01 s, reflects the levels of P700^+^ and PC^+^ (the oxidized forms). The second phase, a slow phase (MR_slow_) resolved in a span of 0.01 to 0.1 s, showed the progressive reduction of P700^+^ and of PC due to the electrons coming from PSII, thus illustrating the connectivity of the electron transport between the two photosystems. Hence, this signal correlates with the PSII functionality and the activity of the electron transport chain between the two photosystems.

Determination of MR in FB1-treated plants is shown in Fig. 7a. The fast initial phase of the MR signal was characterized by a decrease of the MR values in the span from 300 µs to 30 ms, when P700^+^ and PC^+^ presented maximum accumulation. This was followed by a slow (400–500 ms) positive phase caused by the gradual reduction of P700^+^. However, the curves of the leaves treated with FB1 for 24 and 30 h showed a 40% decrease of the MR_fast_ phase compared to the 0 h curve, indicating that the total accumulation of PC^+^ and P700^+^ was affected by the FB1 treatment. Additionally, the MR_slow_ was significantly affected at 30 h by the FB1 treatment. This could be explained by a loss in the efficiency of the electron transport chain to re-reduce the pool of P700 or/and PC upon the actinic illumination. However, plants treated with FB1 after 24 h showed a maximum value of the MR recovery after the fast phase, indicating that the electron transport between PSII and PSI units was still functional but was lost later after 30 h of FB1 exposure.

In the case of the pathogen treatment, the Pst control (0 h) displayed a similar curve to the FB1 control (Fig. 7b). MR_slow_ phase at 10 and 24 h showed a decrease in total amplitude of 40% and 50%, respectively, when compared to the 0 h curve. Contrary to the FB1 curve at 24 h, the Pst curve showed that the re-reduction of P700 after 0.03 s almost completely disappeared. This meant that the electron transport between the two photosystems was impaired, indicating loss of the electron transport capacity between PSII and PSI.

### Chloroplast integrity and ultrastructure are compromised upon FB1 and Pst treatments

**Figure 8:**
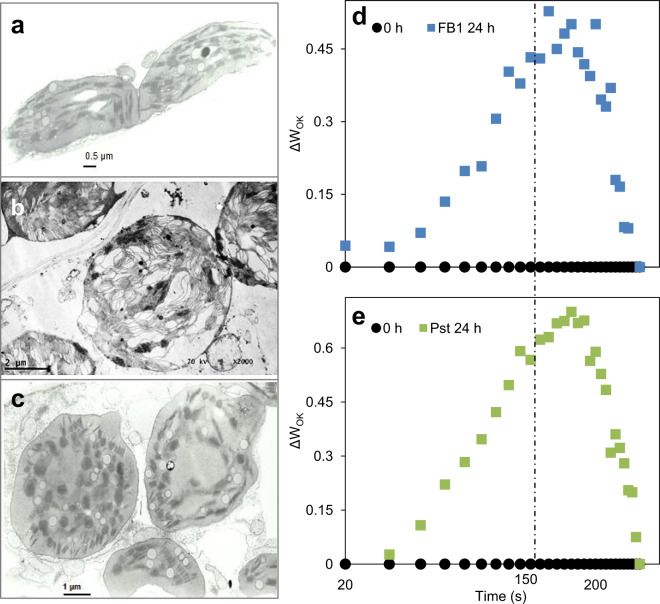
FB1 and Pst induce unstacking and brakeage of thylakoid membranes. Representative transmission electron microscopy images from chloroplasts from leaves after 24 h of *in planta* infiltration with (**a**) 10 mM MgCl_2_, (**b**) 10 µM FB1 or (**c**) 1×10^8^ CFU/ml Pst. Chlorophyll *a* fluorescence L-band (150 µs) (ΔW_OK_) from leaves infiltrated with (**d**) 10 µM FB1 and (**e**) 1×10^8^ CFU/ml Pst. L-Band was obtained by normalizing the fluorescence intensity between O (20 µs) and K (300 µs) steps of the average OLKJIP transient obtained in Fig. 5. Control or 0 h infiltration sample (squares) and 24 h infiltrated sample (triangles) are shown. Curves are representative of three biological replicates.

Changes in photosynthetic functionality is associated with changes in grana stacking^44^. This is manifested by alteration of the efficiency of the electron transport between PSII to PSI^45^, the collapse of the proton gradient^46^, and the impairment of the lateral heterogeneity of the thylakoid membrane components^47^. In order to explore this possibility, the details of the chloroplast structure were investigated by transmission electron microscopy (Fig. 8b,c Supplementary Fig. S3). The control samples showed typical elongated chloroplasts with intact and well stacked thylakoid membranes and very well defined outer and thylakoid membranes (Fig. 8a). In contrast, chloroplasts from the FB1- and Pst-treated leaves (Fig. 8b,c; Supplementary Fig. S3) showed a rounded shape, very highly unstructured grana and thylakoid membranes at different states of disintegration, suggesting that a progressive process of chloroplast destruction was taking place. It was clear that FB1 and Pst treatment induced alterations of the electron transport capacity of PSII that were already detectable at 8 h (Fig. 6) and progressed until a visible macroscopic phenotypical alteration appear.

We calculated the so-called L-band from the average OJIP curves in Fig. 5a–c, as an additional way to confirm that the photosynthetic impairment was caused by alterations in thylakoid ultrastructure. In case of alteration in PSII unit connectivity, a positive band between 20 µs and 300 µs (L-band)^48–50^ should appear in the OJIP transient. Figure 8d,e shows relative changes in fluorescence between 20 µs and 300 µs (ΔW_OK_), where the L-band occurs. In both figures, it was possible to observe a clear positive band at 24 h for FB1 and 18 h for Pst. This confirms the observed disconnection between PSI and PSII (Fig. 6). Altogether, MR, TEM and L-band analysis confirmed that impairment of photosynthetic activity induced by FB1 and Pst led to disruption of thylakoid organization.

## Discussion

In this work, we have studied how two independent treatments that induce PCD elicited by LCBs have the same effect over the function and structure of the chloroplast. Given the role of this organelle on cell function, these results suggest that the chloroplast decay during PCD is an effective and irreversible mechanism that leads to cell death as a defense strategy against non-host pathogens. In detail, we show that the photosynthetic failure is preceded by LCB increase and MAPK activation and involves ROS formation. This indicates that the loss of functionality of the chloroplast is part of the mechanism by which PCD occurs and is one event in the non-host immunity manifested in the HR.

In order to demonstrate the relation between all these events, the experimental framework consisted of a comparison of Pst and FB1 effects on chloroplast function in *Phaseolus vulgaris*. Pst (*Pseudomonas syringae* pv. *tomato*) is a bacterial non-host pathogen for *Phaseolus vulgaris*, while FB1 is an inducer of LCB elevation that leads to PCD^11,16,24^. FB1 has proven to lead to the manifestation of an HR and defense reactions against pathogens^11,16,35^. The comparison between these treatments revealed striking similarities that included same biochemical features (LCB rise, MAPK activation and H_2_O_2_ production), structural changes in the chloroplast (thylakoid unstacking and deformation of the chloroplast) and photosynthetic function impairment. The experimental comparison of both treatments allowed the identification of several pieces of the mechanism underlying plant defense under a scheme of PCD.

In Arabidopsis, FB1 induced an HR-like wound due to a LCB accumulation acting as second messengers^11,16,35^. Here, we also show that FB1 in *Phaseolus vulgaris* induces a LCB rise and downstream events of HR such as MPK activation and ROS production. The parallelism found here for Pst, a non-host pathogen, shows that LCBs are also involved in this type of immune response.  Although, during the non-host immunity response observed in *Nicotiana benthamiana* with *Pseudomonas cichorii*, an increase in gene transcription of SPT subunits was essential to produce HR, LCB changes were undetectable^8^. The successful LCB detection in our case of non-host defense could be due to a difference in LCB extraction and/or to the frequent sampling during the intermittent and irregular LCB increase pattern that contrasts with regular LCB rises induced by pathogens in the PAMPs-triggered immunity (PTI) and in the Effector-triggered immunity (ETI) schemes^51^. Therefore, the LCB surge seems to follow a profile that is specific to the type of immunity. In the case of the PTI, the rise of LCB occurs as a unique, early and short-life peak upon exposure to the pathogen  while in the ETI occurs as a sustained and high LCB surge^51^. It can be suggested that non-host immunity, that contains PTI and ETI features, shows a LCB surge profile that takes elements from both PTI and ETI, which are associated with this  non-host HR. .

MAP cascades are activated upon exposure to several stresses. Based on the  specificity of substrate phosphorylation and antibody reactivity, the present work shows that a MAPK is activated in *Phaseolus* leaves upon FB1, SN and Pst exposure. This result indicates that endogenous and exogenous LCBs are able of eliciting the same response, i.e. the same MAPK activation. In the case of responses to pathogen attack, the most common MAPKs activated are MPK3 and MPK6^37,52^. In addition, the latter has been shown to be the one activated downstream LCB accumulation^11^. Therefore, the molecular mass, the activation by LCB and by SA, and the analogous time response pattern to *Pseudomonas syringae* pv. *tomato* strongly indicate that the MAPK activated is MPK6 as it occurs  in other systems^11^. However, immunoprecipitation with specific antibodies directed against the *Phaseolus* enzyme must settle this point.

It is possible that very low levels of H_2_O_2_ generated at early times and undetected by DAB stain, initiate PCD as part of the initial signaling events that include LCB surge and MAPK activation. Such ROS generation that we were unable to detect has been described as an initial H_2_O_2_ burst  in defense against pathogens, but takes place outside the chloroplast^11,16,36^.

After the signaling steps: LCB increases and MAPK activation, the chloroplast starts a program to stop photosynthesis. The pattern of decay of photosynthesis upon LCBs accumulation was very similar between the FB1 and Pst treatments. Three main characteristics were observed: (1) a decrease in the number of functional reaction centers of PSII; (2) the compromised ability of PSII to reduce the PQ and to sustain the electron transport towards PSI; (3) a loss in the PSII centers connectivity due to severe thylakoid unstacking. These shared features between the FB1 and Pst treatments make us hypothesize that the loss of function of the chloroplast is a well-organized process that has in common LCB surges and MAPK activation.

It is known that during pathogen infections the photosynthetic activity is interrupted^20–22^. The observed effect in this work over linear electron transport and its carriers (as shown by JIP-test and MR) indicates a size decrease of the pool of acceptors. This would lead to an increased amount of ROS accumulation in the chloroplast^53,54^. It is commonly accepted that ROS can be generated in the chloroplast via light sensitization^55–57^ and this could be aggravated by an increase in excitation pressure. This explains why at 24 h, DAB stain detected high levels of H_2_O_2_, which occurred after the first major signs of photosynthesis inactivation started to appear.

Interestingly, it has been reported that PCD induced by FB1 requires light and it has been proposed that ROS produced in the chloroplast could be the second messengers that up-regulate SA synthesis by enhancing PAL activity leading to a PCD HR-like^58^. Also, based only on chloroplast ultrastructure, studies in Arabidopsis, one using FB1^11^ and the other using *Pseudomonas syringae maculicola* 4326 avrRpt2^58,59^ proposed that ROS formed in the organelle may cause disassembly of the photosystems. Altogether, the detected accumulation of H_2_O_2_ in this work must have caused a destructive effect on the photosynthetic ensemble as revealed by the ultrastructural observations.

LCB accumulation produced by FB1 and Pst caused an eventual alteration of chloroplast morphology. It was in the rounded chloroplasts where the large peroxide accumulation occurred and in which thylakoids looked unstacked and broken. In support to this structural and functional damage, the so-called L-band was observed in both FB1 and Pst treatments. The appearance of a positive L-band is attributed to failures in the connectivity between the photosystems^48–50^. For both treatments, the L-band was detected at the times when clear thylakoid unstacking was found. Therefore, the failure of connectivity is reasonably explained by the unstacking of the thylakoid membranes in the leaves exposed to the toxin or the pathogen.

Here we show that during PCD, just after the LCB accumulation and MAPK activation took place, a decrease in the chloroplast electron carriers occurred. This was followed by a large accumulation of ROS inside the chloroplast that lead to collapse the thylakoid membrane system. It is possible that ROS could catalyze the oxidation of the thylakoid membrane lipids. This is supported by the formation of H_2_O_2_ inside the chloroplasts^17^ and by the promotion of extensive chloroplast damage by FB1, that includes the degradation of proteins from the chloroplast stroma^57^. Degradation of core proteins from the photosynthetic complexes has been reported but in a model that involves a compatible plant-pathogen interaction^59,60^. In nature, several scavenging processes ensure a low concentration of ROS inside the chloroplast^61^. Therefore, an intermediary process is required to allow accumulation of ROS in the chloroplast; thus, a midway step between accumulation of LCBs and inactivation of the chloroplast would be required to shut down ROS scavenging processes. The decay of the photosynthetic machinery observed can be related to a silencing of the chloroplast protein expression^62,63^ associated with an absence of the repair mechanism that produces the photodamage of PSII^64–67^. Therefore, the damaged photosystems would not be replaced by new ones^68^ and the chloroplast would produce ROS via antenna sensitization due to excitation pressure^69^.

## Conclusions

We have shown that two agents inducing PCD by accumulation of LCBs led to the organized decay of the chloroplast function and structure. This is characterized by the decline of the light reactions of photosynthesis, which leads to the accumulation of ROS in the chloroplast. Thus, the observed HR-PCD involves signaling and executory steps that include the rise of LCBs, MAPK activation, collapse of both photosystems, massive ROS production and structural disintegration of the thylakoid membrane and the chloroplast. This orchestrated and programmed sequence of events leads the plant cell to its self-destruction as a defense strategy against dissemination of biotroph pathogens. The features of such molecular events may vary in other cases of PCD or in other forms of immunity but because of  their impact on cell energetics, they seem to function as a stratagem to reach a point of no-return in the program of cell death in immunity.

## Supplementary information

Supplementary information1

Supplementary information2

Supplementary information3
