# Supplementary material for: Organized Disassembly of Photosynthesis During Programmed Cell Death Mediated By Long Chain Bases
Source: Sci Rep. 2020 Jun 25;10:10360. doi: 10.1038/s41598-020-65186-8 (PMC7316715; doi:10.1038/s41598-020-65186-8)
Supplement: Supplementary file 1 — Supplementary information1 [file 41598_2020_65186_MOESM1_ESM.pdf]

SUPPLEMENTARY INFORMATION 1

ORGANIZED DISASSEMBLY OF PHOTOSYNTHESIS DURING PROGRAMMED CELL DEATH MEDIATED BY LONG CHAIN BASES

Alonso Zavafer, Ariadna González-Solís, Silvia Palacios-Bahena, Mariana Saucedo-García, Cinthya Tapia de Aquino, Sonia Vázquez-Santana, Beatriz King-Díaz and Marina Gavilanes-Ruiz*

Fig. S1

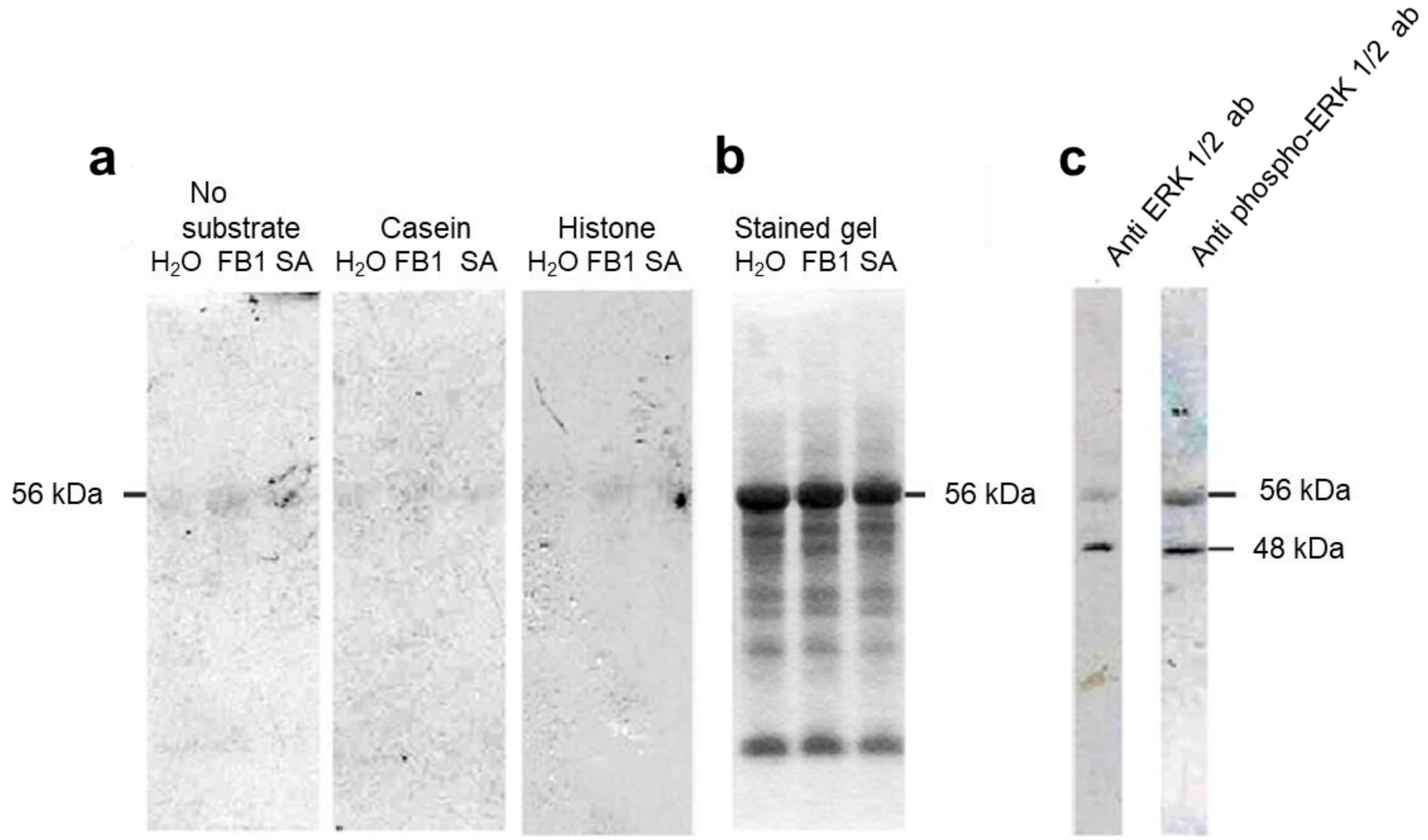

Figure S1. MAPK assay specificity and MAPK reactivity to anti phospho-ERK. (a) Specificity of the MAPK assay. Phaseolus leaves were exposed to 40 µM FB1 and 1 mM salicylic acid (SA) and extracts were obtained and used in an in-gel kinase assay using no substrate (H_2O) or casein or histone in the gel matrix. (b) The respective samples were stained with Coomassie blue. (c) Immunoprecipitation with antibodies anti-ERK and anti-phospho-ERK. Crude extracts from Phaseolus leaves were prepared and tested for immunoprecipitation analysis using antibodies anti-ERK1/2ab and anti-phospho-ERK1/2ab and assayed in an in-gel assay as described in Material and Methods. The masses of protein kinases in kDa are indicated. Images are representative of at least three independent biological replicates.
