# Supplementary material for: Organized Disassembly of Photosynthesis During Programmed Cell Death Mediated By Long Chain Bases
Source: Sci Rep. 2020 Jun 25;10:10360. doi: 10.1038/s41598-020-65186-8 (PMC7316715; doi:10.1038/s41598-020-65186-8)
Supplement: Supplementary file 2 — Supplementary information2 [file 41598_2020_65186_MOESM2_ESM.pdf]

SUPPLEMENTARY INFORMATION 2

ORGANIZED DISASSEMBLY OF PHOTOSYNTHESIS DURING PROGRAMMED CELL DEATH MEDIATED BY LONG CHAIN BASES

Alonso Zavafer, Ariadna González-Solís, Silvia Palacios-Bahena, Mariana Saucedo-García, Cinthya Tapia de Aquino, Sonia Vázquez-Santana, Beatriz King-Díaz and Marina Gavilanes-Ruiz*

Fig. S2

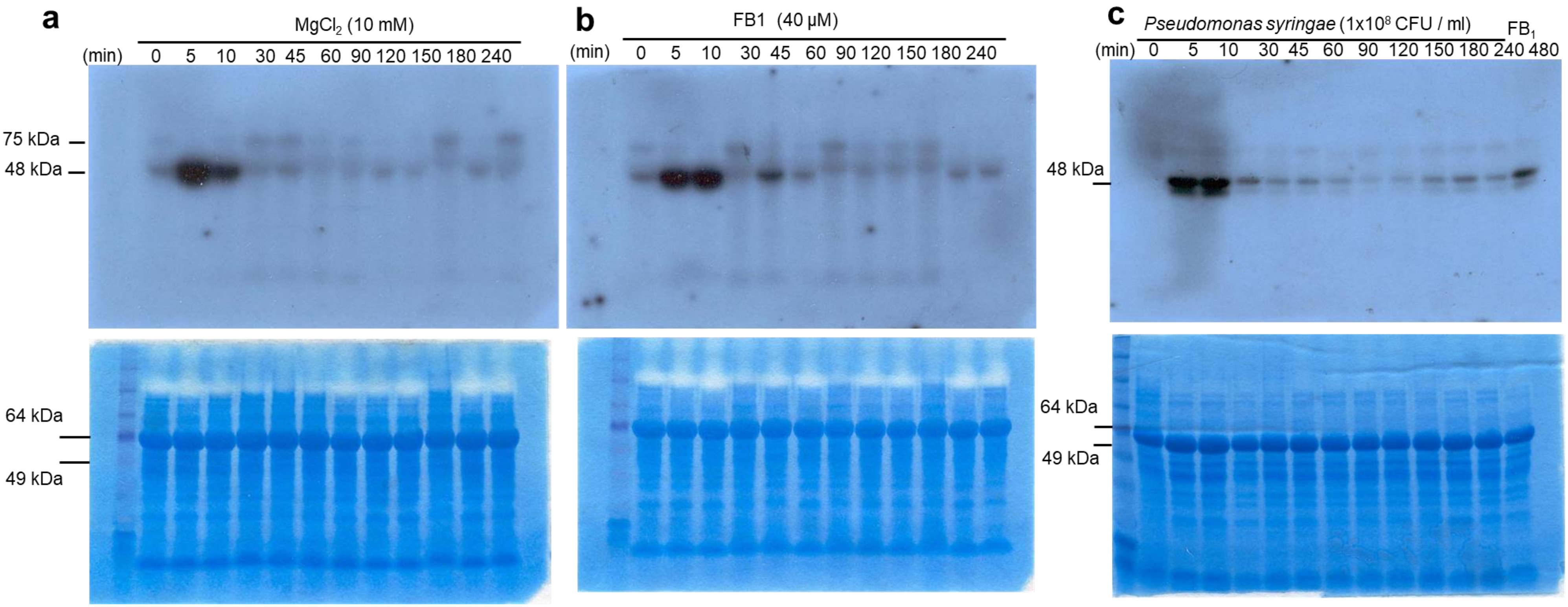

Figure S2. MAPK activation is induced by FB1, Pst, sphinganine and salicylic acid in Phaseolus leaves. Leaves were infiltrated *in planta* with (a) $MgCl_2$, (b) FB1, (c) Pst, (d) sphinganine (SN), and (e) salicylic acid (SA) at the concentrations shown. At the indicated times, infiltrated leaf sections were cut and soluble protein fractions were obtained to determine in-gel MBP phosphorylation to estimate MAPK activity. SN was dissolved in 0.05 % Silwet L-77 (SW) and SA was at pH 7.0. Upper parts of the panels show the complete autoradiographies from the in-gel activities. Lower parts of the panels show the correspondent complete Coomassie blue stained gels. Experiments are representative of at least three biological replicates. St, molecular mass standards. (It continues in next page).

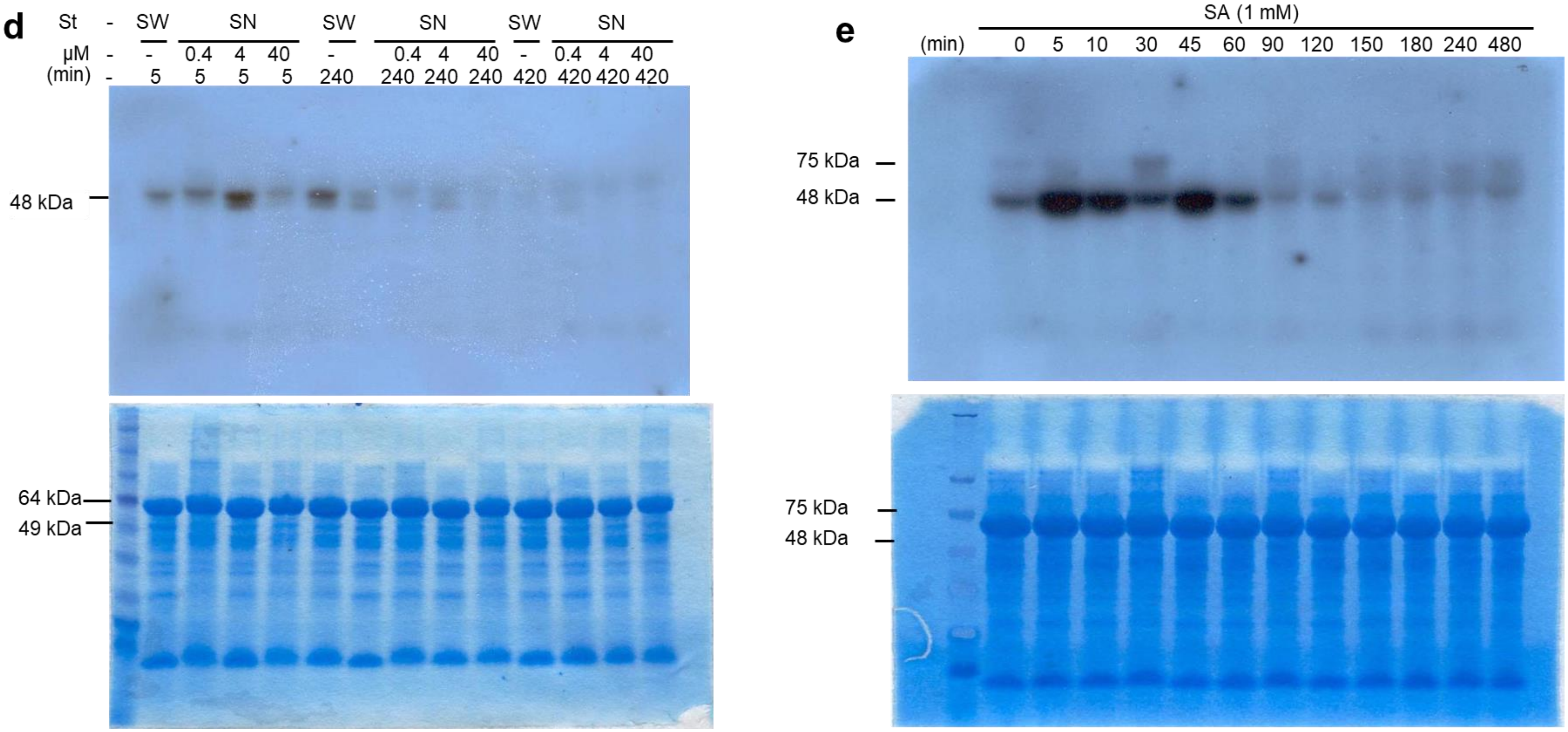

Figure S2. MAPK activation is induced by FB1, Pst, sphinganine and salicylic acid in Phaseolus leaves. Leaves were infiltrated *in planta* with (a) $MgCl_2$, (b) FB1, (c) Pst, (d) sphinganine (SN), and (e) salicylic acid (SA) at the concentrations shown. At the indicated times, infiltrated leaf sections were cut and soluble protein fractions were obtained to determine in-gel MBP phosphorylation to estimate MAPK activity. SN was dissolved in 0.05 % Silwet L-77 (SW) and SA was at pH 7.0. Upper parts of the panels show the complete autoradiographies from the in-gel activities. Lower parts of the panels show the correspondent complete Coomassie blue stained gels. Experiments are representative of at least three biological replicates. St, molecular mass standards.
