# Supplementary material for: Organized Disassembly of Photosynthesis During Programmed Cell Death Mediated By Long Chain Bases
Source: Sci Rep. 2020 Jun 25;10:10360. doi: 10.1038/s41598-020-65186-8 (PMC7316715; doi:10.1038/s41598-020-65186-8)
Supplement: Supplementary file 3 — Supplementary information3 [file 41598_2020_65186_MOESM3_ESM.pdf]

SUPPLEMENTARY INFORMATION 3

ORGANIZED DISASSEMBLY OF PHOTOSYNTHESIS DURING PROGRAMMED CELL DEATH MEDIATED BY LONG CHAIN BASES

Alonso Zavafer, Ariadna González-Solís, Silvia Palacios-Bahena, Mariana Saucedo-García, Cinthya Tapia de Aquino, Sonia Vázquez-Santana, Beatriz King-Díaz and Marina Gavilanes-Ruiz*

Fig. S3

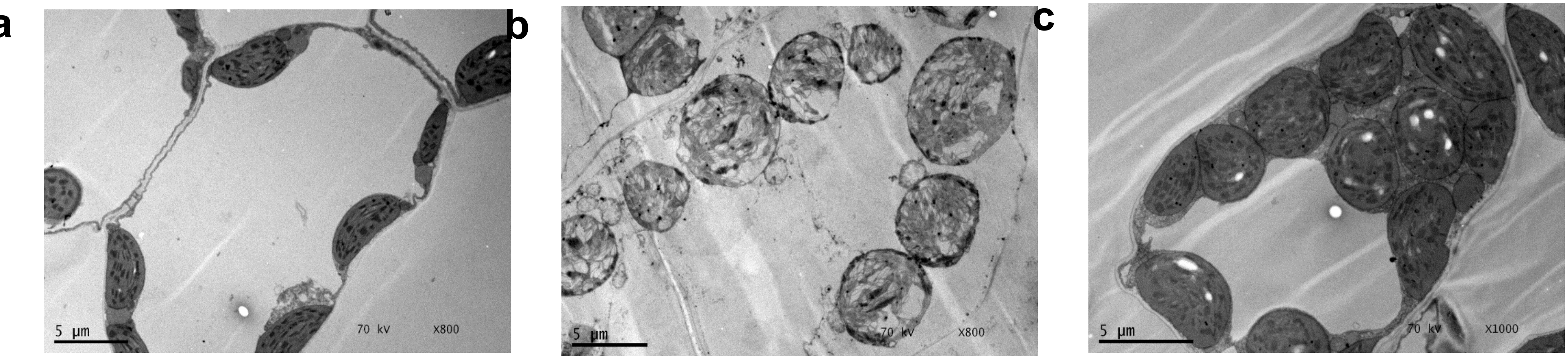

Figure S3. Effects of FB1 and Pst treatments on the chloroplast morphology. Phaseolus leaves were infiltrated *in planta* with (a) 10 mM $MgCl_2$, (b) 10 µM FB1 or (c) 1x 10^8 CFU/ml Pst. Sections from the leaves were taken after 24 h, fixed and treated for analysis by transmission electron microscopy as described under Materials and methods. Representative images from at least three independent replicates are shown.
